# Around the Hospitals

**Published:** 1895-11-02

**Authors:** 


### AROUND THE HOSPITALS.

[NOTICE TO THE MANAGERS OF INSTITUTIONS.—Special space is now reserved for the insertion of notices of hospital meetings and festivals. The Editor requests that all notices may reach THE HOSPITAL Office, 428, Strand, at least one week before the date of each meeting.]

**The Royal Mineral Water Hospital, Bath.**—This hospital confers great benefits on numbers of poor patients from all parts of the kingdom. It is intended only for those whose ailments are especially open to alleviation by means of the waters of Bath, and who could not otherwise secure their benefit. Full opportunity is given for recovery, as the average residence of patients is forty-five days. The income from investments amounts to £3,199, the average expenditure to £5,108; consequently over £2,000 has to be provided annually. Private subscriptions last year equalled £648, and those through the Poor Law, through which patients are received, to £785; donations and collections to about £400; so that, had it not been for legacies, the accounts would have shown a balance on the wrong side. Considering the usefulness of the institution, larger contributions from the localities whence the patients are drawn might well be looked for. The expenditure in the last year was a high one, owing to improvements in the sanitary arrangements of the hospital which were effected, but an allowance for necessary repairs and improvements must be made in most years. The number of patients received during the year was 1,422; the large majority of these were suffering from rheumatic affections and gout.

**Miller Hospital, Greenwich.**—Owing to the locality in which this institution is situated, a very large number of the cases treated arise from accidents. The accommodation is no longer sufficient to meet the requirements of the neighbourhood. An appeal for funds to extend the premises is being made. The hospital excites a great deal of interest locally, and a variety of entertainments are held in its benefit. The report of the hospital, whilst it deals exhaustively with the nature of the cases received, and gives full particulars as to subscriptions, does not give the information necessary to form a judgment of the economy of management by stating the cost per patient and per occupied bed. By studying the accounts it will be seen that the cost of maintenance is large. This is especially observable in the items of malt liquors, wines, and spirits. During the year 283 in-patients and 3,909 out-patients were received. The ordinary income amounted to £2,364, and the ordinary expenditure to £3,121.

